# Exploring the interplay of interpersonal and contextual dynamics in youth sports injuries: a comprehensive narrative review

**DOI:** 10.1136/bmjsem-2024-001964

**Published:** 2024-07-16

**Authors:** Christian Thue Bjørndal, Solveig Hausken-Sutter, Merete Møller, Grethe Myklebust, Hege Grindem

**Affiliations:** 1 Department of Sport and Social Sciences, Norwegian School of Sports Sciences, Oslo, Norway; 2 Child and Youth Sport Research Center, Norwegian School of Sports Sciences, Oslo, Norway; 3 Oslo Sports Trauma Research Center, Norwegian School of Sport Sciences, Oslo, Norway; 4 Department of Sports Science and Clinical Biomechanics, University of Southern Denmark, Odense, Denmark; 5 Department of Sports Medicine, Norwegian School of Sport Sciences, Oslo, Norway

**Keywords:** Adolescent, Sociology, Sporting injuries, Sport and exercise psychology, Intervention effectiveness

## Abstract

Injuries are recognised in sports and exercise medicine as not isolated incidents but complex outcomes. This is because an athlete’s health trajectory is understood to be shaped by dynamic, complex linkages between individual performance, biology, and the wider social and cultural contexts and systems in which individuals perform. Despite this recognition, little attention has been paid to how interpersonal and contextual dynamics can potentially affect the risk of injury by influencing the choices and decisions made by coaches, parents and athletes. To address this gap, this narrative review bridges insights from sociocultural studies in sports with the findings of sports injury research. The narrative review aims to identify and summarise how interpersonal and contextual dynamics influence the risk of youth sports injuries. The results reveal the pressures faced by athletes, often leading to compromised health. Moreover, the review underscores the importance of designing complex interventions and strategies to promote healthier practices in youth sports. Specifically, intervention programmes should prioritise raising awareness of injury risks, cultivating effective communication skills and fostering supportive training environments.

**WHAT IS ALREADY KNOWN ON THIS TOPIC**

- Youth sports injuries are complex and influenced by the multifaceted and dynamic interplays between interpersonal, biological, psychological and contextual factors.
- Qualitative methodologies can provide insight into the contexts in which injuries occur and what contextual factors influence injury development.
- There is a need for complex interventions to be developed that are tailored to real-world settings and are better at preventing injuries in youth athletes.

**WHAT THIS STUDY ADDS**

- The present review provides an overview of contemporary literature on interpersonal and contextual studies of youth athletes’ experiences related to sports injuries.
- Interpersonal and contextual dynamics influence youth sports injuries, place pressure on athletes, and lead them to compromise their health.
- This pressure often results in athletes, coaches and medical staff making inappropriate decisions and engaging in harmful behaviours.

**HOW THIS STUDY MIGHT AFFECT RESEARCH, PRACTICE OR POLICY**

- This study demonstrates the need for injury prevention interventions that foster positive coaching practices and athlete well-being.
- When developing complex injury prevention interventions, scholars should incorporate comprehensive, contextual and inclusive qualitative methodologies with a sociocultural focus.

## Introduction

 Regular participation in youth sports offers a broad range of physical, mental and social benefits.^1 2^ However, such engagement also comes with challenges, primarily due to the risk of sports-related injuries.^3^ In Norway, acute and overuse injuries are found among 43%–53% of youth athletes. Of these injuries, 35%–38% are categorised as substantial, and a higher prevalence among female athletes has also been noted.^4 5^ The consequences of these injuries extend beyond their physical impact because they also contribute significantly to wider systemic impacts, such as high sport drop-out rates among youth athletes.^6^

Sports and exercise medicine researchers recognise that injuries are not isolated incidents but result from individual actions and choices. Instead, they are seen as the outcomes of complex interplays between individuals, their physical environment, and the wider social environment.^7^ Intricate, multifaceted and dynamic interactions between interpersonal, biological, and psychological systems and broader contextual factors affect athletes’ health throughout their careers.^8^ Particularly in the context of youth sport, interpersonal and contextual dynamics influence athletes’ susceptibility to injuries, their response to injuries, and their recovery processes.^9^ Understanding these dynamics and their impacts is crucial for developing strategies to prevent injuries among young athletes.

Complex interventions can encompass healthcare or social interventions with multiple interacting components and interventions tailored to the delivery context.^10^ There is a growing recognition, therefore, that complex interventions for injury prevention should be informed by the specific context and circumstances in which they are located, as well as by the substantial influence of stakeholders’ (eg, coaches, parents and athletes) decision-making processes and lived experiences.^11 12^ Despite this recognition, notable gaps remain in understanding how interpersonal and contextual dynamics shape the experiences and decisions of athletes, and how these may contribute to a higher risk of injury.^13^

Sociocultural investigations of sport and exercise explore the interpersonal and contextual dynamics that shape individuals’ thoughts, feelings and behaviours within sports settings.^14^ These qualitative studies examine how societal norms, cultural beliefs, historical contexts and social structures impact various aspects of sports participation. They also examine athletes’ experiences and decisions related to injuries^15^ and offer valuable insights into injury risk factors.

In contrast, sports injury prevention intervention research focuses notably different^16^ and is preoccupied predominantly with injuries to specific body parts. Injury definitions are based on objective measures, such as medical treatment or time loss from training and/or matches.^17^ While such biomedical injury definitions provide valuable data on objective injury outcomes, they are limited in accounting for the broader sociocultural context in which such injuries occur. Sociocultural studies of sport, in contrast, investigate the subject of injuries but also encompass broader concepts that extend beyond empirical, epidemiological injury definitions.^18^

This contrast in investigative focus between sociocultural studies and sports injury prevention intervention underscores the need for greater integration and collaboration between these two areas of research. Doing so, we contend, could help to develop more comprehensive and effective injury prevention strategies that consider both the biomedical and sociocultural dimensions of sports participation.

We, therefore, conducted a narrative review of the literature on interpersonal and contextual studies of youth athletes’ experiences related to injuries in youth sports. We have highlighted key insights from qualitative sociocultural research in sport. The two objectives of the study were (a) to identify and summarise how interpersonal and contextual dynamics influence the risk of youth sports injuries and (b) to discuss the implications of these findings for developing complex interventions that can foster healthier practice in youth sports.

### Theoretical framework

**Figure 1:**
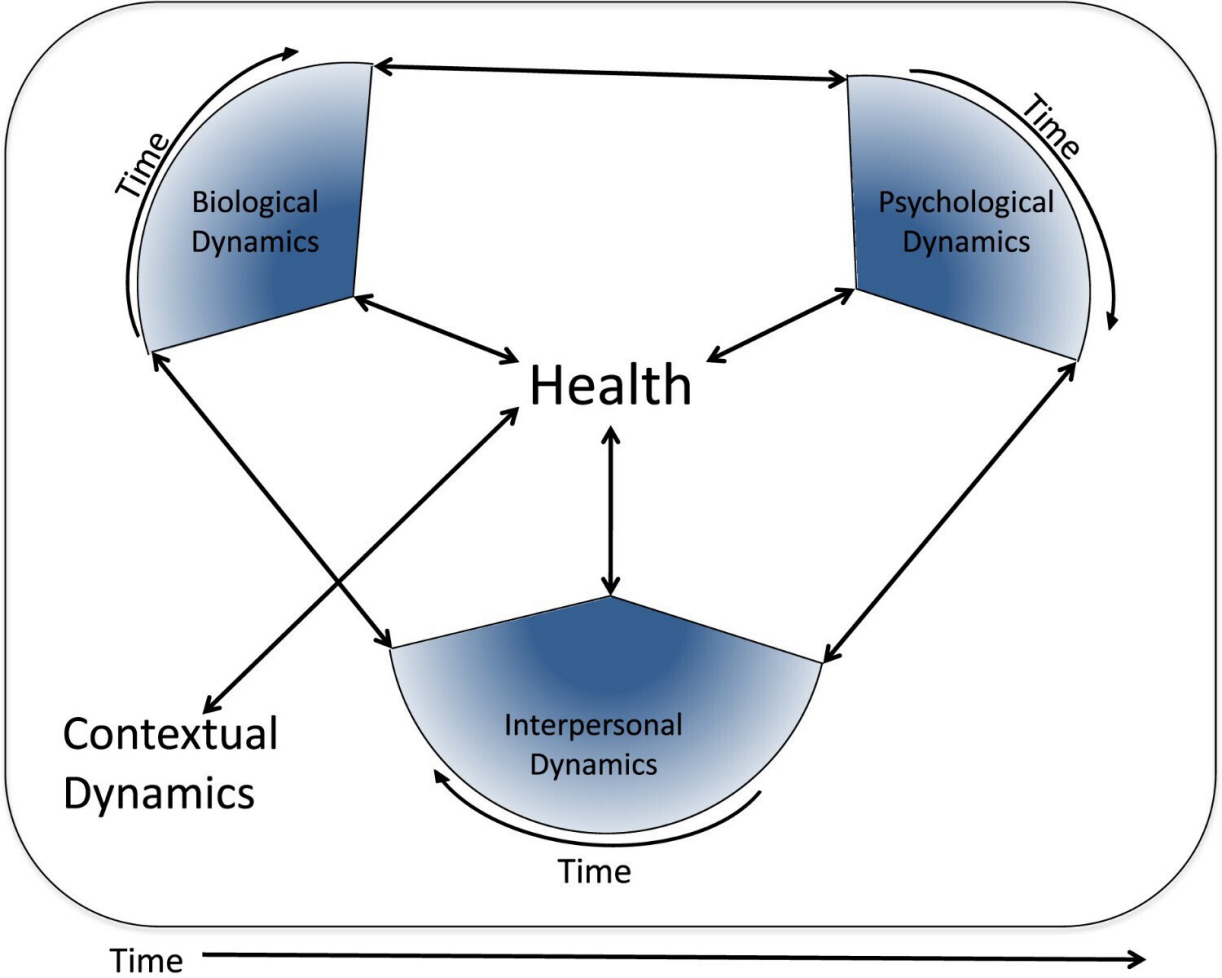
The dynamic biopsychosocial model of health. This model expands on the biopsychosocial model by incorporating a dynamic systems perspective and further clarifying social influences by applying Bronfenbrenner’s theories of development. Each model component includes systems that reciprocally influence other model dynamics and affect health. Social dynamics are divided into interpersonal factors and broader contextual dynamics (eg, culture). Additionally, all dynamics change over time, and individual systems may ebb and flow in their impact on the individual’s health (this ebb and flow is termed centrality and is represented by the blue shading in each wedge). The darker portions of the wedge include more central factors for the individual. Reprinted with permission from Lehman *et al.* Copyright 2017 John Wiley and Sons.^8^

The presentation and analysis of the findings in this study are based on an adaptation and refinement of the dynamic biopsychosocial model of health developed by Lehman *et al*^8^ to facilitate the organisation and synthesis of the findings more comprehensively and effectively (figure 1). The dynamic biopsychosocial approach was selected because of its comprehensiveness and integrative approach to understanding health outcomes—an approach that makes it particularly suitable for youth sports injury research.

**Figure 2:**
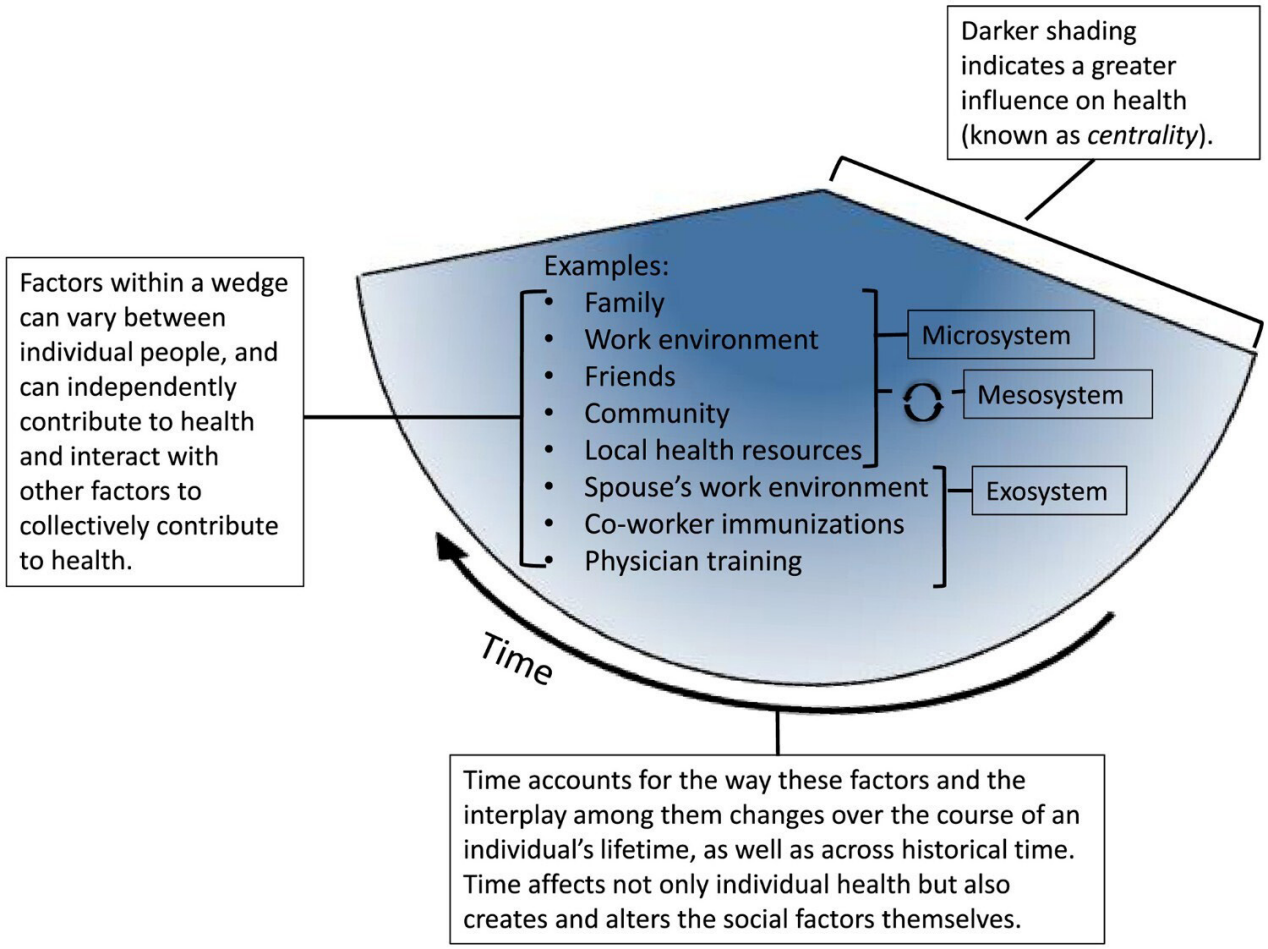
A close‐up example of interpersonal dynamics. Bronfenbrenner’s classic terminology is applied to the domain of health as a way to further elaborate interpersonal factors in the dynamic biopsychosocial model of health. Each wedge of the model is influenced by factors that may change and mutually affect each other over time. Reprinted with permission from Lehman *et al*. Copyright 2017 John Wiley and Sons.^8^

Interpersonal dynamics (figure 2) in this model include the effects of *actual* and *perceived* social contacts on health.^8^ These interpersonal dynamics are understood to be reciprocally influenced by factors at a microsystem, mesosystem and exosystem level.^19^ The first level, namely the microsystem, refers to the immediate environment in which an individual interacts regularly. Components at this level typically include the people within an individual’s family, school, peer group, neighbourhood and other immediate settings where a person interacts directly with others. Coach support for athletes, for example, is an important dynamic that has been shown to help athletes cope with injuries (eg, through providing emotional support, encouragement and motivation, information and guidance, adaptation of training and support).^20^

Mesosystem dynamics include *interactions* among microsystem elements such as the home, school and sports environment(s). Exosystem dynamics encompass the interactions and influences between individuals and settings that are not directly experienced by individuals but still impact their development and experiences. According to the model, the growth and behaviour of individuals are embedded in and influenced by multiple interconnected systems. For example, the knowledge conveyed through the coach’s education can profoundly influence the training methodologies that coaches apply, subsequently influencing athletes’ learning and development.

Contextual dynamics in the dynamic biopsychosocial model of health occur at a macrosystem level and reflect broader patterns of shared culture, norms, policies and values.^8^ For example, prevailing cultural values can significantly affect an individual’s perception of pain, sickness and health-related complaints. In some settings, for example, the occurrence of injuries and the acceptance of the inherent health risks of sports may be normalised; in others, they may not.^21^ These dynamics help shape interpersonal, psychological and biological factors and are, in turn, shaped by them.

These different system levels serve as a heuristic for conceptualising and structuring sociocultural influences on injury development. However, in reality, they are intertwined and dynamic rather than separate.

## Methods

We conducted a narrative review of literature for its suitability for synthesising and evaluating a critical analysis of a diverse body of literature,^22^ drawing on guidelines for writing narrative reviews outlined by Green *et al*.^23^ As such, this methodology was appropriate for assessing the current knowledge on the interplay between interpersonal and contextual dynamics and youth sports injuries.^23^

### Data collection

We used a three-phase multifaceted search strategy to ensure a comprehensive coverage of the literature on interpersonal and contextual dynamics relevant to youth sports injuries. First, we conducted an extensive literature search across the SPORTDiscuss, Web of Science, PsycINFO and PubMed databases. The search terms included various combinations of “youth”, “adolescent”, “sport”, “injury”, “overuse”, “pain”, “context”, “social”, “culture” and “qualitative”. The second author conducted the initial search. The first and second authors independently performed (a) title screening, (b) abstract screening and (c) full-text screening.

The first and second authors determined whether the studies focused on youth sports aged 10–19 years, assessed whether they employed a qualitative methodology, and examined whether the studies specifically addressed injury. After the first and second author independently screened the title, abstract and full text, these two researchers discussed and determined which studies identified in the search should be included or excluded. In cases where conflicting decisions arose during the screening process, the first and second authors met to discuss and reconcile the conflicting differences. They made a final decision about including or excluding the article. After selecting the relevant articles, the first and second authors closely read the full-text versions.

During the second data collection phase, we conducted a cited reference search of the articles identified through the primary search method. This secondary search method allowed us to uncover additional relevant publications and expand our review’s scope.

In the third phase, we sought to maximise the retrieval of relevant sources by complementing the above-described search with literature already known to the authors based on the initial inclusion criteria.

Our focus during the data collection centred on investigating young athletes’ injuries-related experiences. To ensure the rigour and relevance of our review, we excluded book chapters, reports, and conference papers that had not been peer-reviewed. Non-English language publications were omitted because the project’s budget could not cover the expense of translation. Additionally, we limited our scope to peer-reviewed studies published after 1 January 2000.

We used a theoretically driven analytical approach to analyse our empirical data. This was grounded in our chosen theoretical framework of the dynamic biopsychosocial model of health.^24^ Our approach facilitated the identification of themes and patterns within the qualitative data. It enabled us to describe and interpret their significance within the context of our research objectives.

## Results

A total of 15 studies were included in the review.^25^,^39^ Overall, the predominant focus of the studies was on sports in European countries.^25^,^3236 37 39^ Three studies were conducted in North America,^33 34 38^ and one in Oceania.^35^ The participant composition of the studies varied: eight only included female participants,^25^,^3034 39^ one included only male participants^33^ and six incorporated a mix of female and male participants.^3132 35^,^38^ The size of the participant cohorts ranged between 1 and 24 athletes. The studies employed diverse research methodologies, including individual and group interviews,^25^,^2831 35^ as well as ethnographic methods and participant observation.^2930 32^,^34^ Details about the purpose of each study, the units of analysis and the key findings are presented in online supplemental material 1.

Our analysis categorised the studies into two main groups, ‘interpersonal dynamics’ and ‘contextual dynamics’ (see online supplemental material 2 for more information). In the ‘interpersonal dynamics’ category, the coach–athlete relationships significantly influenced athletes’ tendencies to push through injury and pain. In the ‘contextual dynamics’ category, the sport’s ethos, rules and norms strongly influenced athletes’ habitual responses to pain and injury.

### Interpersonal dynamics influencing the risk of injuries

Seven studies provided significant insights on the interpersonal dynamics within athletes’ immediate environment that influence the risk of injuries.^25^,^2830 31 35^ Overall, coaches had a substantial influence on the health of athletes, often leading them to push through injury and pain. Podlog *et al*’s^35^ study of the experiences of youth athletes recovering from injuries noted the direct influence of coaches on athlete perceptions of return-to-sport experiences. It included examples such as athletes themselves reporting that they were ‘trying harder [in rehabilitation] because [the] encouragement from … [their] coach was motivating’.^35^

Barker and Bailey^25^ examined the cumulative life events in a 19-year-old female athlete and how her relationship with her coach influenced her engagement in distance running. Their study illustrated the power coaches wield in shaping the identities and experiences of those they coach. This particular coach–athlete relationship resulted in the athlete becoming dependent on being told what to do and led to habitual compliance with her coach’s instructions. Although this resulted in numerous injuries, the athlete pushed through injury and pain as she feared that the coach would not respect her as an athlete:

I told Dave about a knee injury. I had told him I was in pain, as he always has a go if you don’t tell him before the session. But I always feel like I have to push through an injury for fear that he won’t respect you as an athlete. I never have an issue with pushing, but then part of me feels he should be the one telling me to stop. At no point did he say stop, so I carried on.^25^

Athletes sometimes went well beyond simply pushing through injury and pain. In Barker-Ruchti and Schubring’s^26^ study of the experiences of a female elite gymnast transitioning in and out of the sport between the ages of 6 and 15 years, for instance, a difficult relationship between the coach and the athlete led, ultimately, to self-inflicted injury:

Upon returning from the second competition, Marie wanted to go home. Her coach, however, had other plans and ordered Marie to continue training. At that point, Marie could only see one way out: being injured. In her first training session, Marie “hurt” her ankle, which once a medical practitioner confirmed her inability to train, allowed her to leave [the training camp].^26^

This study, in particular, highlighted the enduring influence of coaching practices on young athletes and underscored the importance of coaches recognising the value of supportive sports relationships and environments and the potential health costs of not doing so.

Kuhlin *et al*’s^30^ study of the long-lasting impact of a coach-athlete relationship on a former female figure skater showed how an athlete’s self-investment could create a relationship characterised not only by the trust but also by dependence and obedience. In this instance, the complex interconnection between the coach and the athlete meant that the athlete could not realise the severity of her injury or the detrimental impacts it was having on her health, well-being, personal development and sporting career. For example, she unquestioningly worshipped her coach, even when met with dismissive responses like a smirk and eye roll, minimising her absence from training due to illness and a knee injury as a mere exaggeration. She noted:

If I am sick or injured, will she [the coach] think that I am not dedicated enough? My coach is probably right about Michaela [another skater in her training group]. If she wanted to become a figure skater, she should be here.^30^

Importantly, athlete behaviour and decision-making processes related to injury management are not only influenced by coaches. One study of young German athletes’ experiences in elite sports highlighted specific instances in which physicians also affected the health of athletes by downplaying the severity of the athletes’ injuries and encouraging them to return to sports prematurely.^31^ Effectively, this meant that the athletes in the study were made to prioritise short-term performance over long-term health considerations, particularly during competitions:

But I still did some competitions then [when her body was hurting], including youth championships. I did it with shots. So there the doctor gave me a shot. And then I could do stuff for two days and then, after that, nothing again. So then, before quals, Jason [the supervising physician] injected me. Just at youth championships, because when we were abroad another doctor injected me. And he had arranged with Jason, somehow, what the shot was and when and so on.^31^

Athletes are also affected by the wider dynamics between sports, school and home settings within the mesosystem. These dynamics can prompt athletes to make inappropriate compromises related to their injuries and to become more tolerant of enduring pain. For example, the (in)appropriate balance between sports and academic requirements and leisure time and the (insufficient) coordination across different contexts such as clubs and sports and school training and competitions may influence how youth athletes experience pain. Bjørndal and Ronglan^28^ have shown that well-functioning coordination between different practice settings appears to be central to successful long-term talent development. However, a breakdown in coordination may lead to insufficient recovery and can, in turn, increase the risk of longer-term injuries. Bjørndal *et al*’s^27^ study of the transition from junior to elite handball in Norwegian female handball provided further examples of how injuries occurred from inadequate support, notably from coaches and medical staff:

Nobody took me seriously. I said that [the injury] hurt. … When I got back home, we were on a pre-season training camp and there [the coach] did not take me seriously. I told him that it hurt but he commanded me to run high-intensity intervals with the rest of the team. When I came back home I was totally wrecked.^27^

In this study, repeated injuries were caused by factors such as the extensive number of activities across the talent development context, the high-risk nature of competitive handball, and the coaches being unable to mutually and sufficiently adapt to each other’s constraints.^28^

### Contextual dynamics influencing the risk of injuries

Eight included studies provided significant insights into the wider contextual dynamics that influence the risk of injuries in youth athletes. The impact of the ethos, rules, norms and expectations of sport on youth sports injuries emerged as a key theme.^2932^,^34 36^

In a study exploring perceptions and experiences of injury by Swedish adolescent athletes across multiple sports, a male participant expressed in a focus-group interview that injuries had become normalised in competitive sports: ‘Everybody gets injured, and you have to accept the injury to be able to continue with sports participation’.^37^ The authors interpreted this normalisation as a coping strategy to adapt to a new situation, such as getting an injury. Athletes in the study learnt, for example, to adapt and to behave in ways that compromised their health, such as perceiving pain as a natural part associated with normal sports participation, and consequently became socialised into accepting pain. Similar findings were echoed in a 2016 ethnographic study exploring the psychosocial influence of sports culture on overuse injuries in Italian rhythmic gymnastics. The study illustrates how athletes adopt and embody sociocultural values and norms such as self-discipline, commitment and responsibility. This was evident in the display of ‘mental toughness’, where athletes accepted pain as part of the sport and continued training despite discomfort:

The pain starts shooting up through the left side of my back, as if wanting to prove who is stronger, urging to stop me. I remind the pain that I am a gymnast, and I’ll do what any ‘good’ gymnast would do: I [will] grit my teeth and smile.^29^

In another study conducted by Fenton and Pitter, 15 to 18-year-old male rugby players were also found to be strongly influenced by their coaches, peers and medical staff:

Just like in games, if I jam my fingers or break my fingers, I’ve broken fingers and still had to play in the game; that’s something if you still feel you can still play you have to keep it down so you don’t get pulled off.^33^

Such studies indicate that athletes are expected to disregard and minimise pain and that doing so is not only common but also expected. They indicate, too, that tolerating pain is almost a socially accepted expectation. One 15-year-old female aesthetic sport athlete in the study by Thiel and colleagues stated:

At the end of the day it is like THAT: In competition, nobody is interested in whether you suffer pain or not. You don’t get extra points for clenching your TEETH. Therefore you don’t tell it to anybody or SHOW it but you try to play (laughs) the HARD guy.^36^

The studies of Fenton and Pitter^33^ in Canadian male rugby and Thiel and colleagues^36^ across multiple German sports demonstrate how athletes’ injuries are frequently caused by a disconnection between athletes’ physical capabilities and the demands imposed on them by the broader sporting culture in which such sports are located. These include social norms and risk-taking behaviours learnt from other players and coaches. These studies also suggest that such conflicting demands force athletes to make difficult or inappropriate health decisions. Similarly, conflicting pressures and demands were evident in Schubring and Thiel’s^32^ study of the growth experiences of adolescent athletes in German elite sport. In their interviews with athletes, the authors noted that the combination of factors, such as an increase in training load and ignoring pain, overloaded athletes’ physical capacity. One handball player, for example, experienced severe knee problems after she was transferred to a sports academy. In her new environment, she faced more intense training loads and a culture that normalised the concealment of pain. As a result, she felt guilty for not participating in training sessions despite her injury:

That’s a tremendous change [in training load] and my knees were already not that healthy before and now they are damaged. … Well, in the beginning, it didn’t make a difference to me—it still hurt but I played because I absolutely wanted to play. That’s it too. My ambition, that’s really quite dumb, because I always want more than I should.^32^

The studies we identified included many examples of athletes pursuing elite sporting careers. However, similar contextual dynamics revealing cultural norms and values have been reported across many other age groups and performance levels. A 3-year ethnographic study conducted by Malcom^34^ (working as an assistant coach for a softball team) noted that adolescent girls entering recreational softball gradually conformed to the norms of the sport and coaching environment. In this environment, the girls learnt to cope with pain and injuries by ‘shaking them off’ and ‘toughing them out’ (ie, disregarding them). Significantly, Malcom^34^ observed that it was the coaches themselves who promoted this behaviour of disregard and even ‘occasionally glorifying pain’ by ignoring the girls’ complaints, making jokes when the athletes experienced pain and telling the girls to ignore minor injuries. The girls, as Malcom noted, learnt to conform to these expectations and modelled their responses to pain and injuries on those demonstrated by the coaches:

“Fire it home! Come on, throw it in here!” Liz threw the ball hard, and it landed in Joe’s hand with a smack. With a smile on his face, Joe said loudly, “That’s it! THAT HURT. Good throw!” When Joe caught their hard throws in his bare hand without flinching, he demonstrated his “toughness” as a ballplayer and modelled the sports ethic to the girls on the softball team.^34^

Malcom’s^34^ study demonstrates how the socialisation process of young athletes in competitive sports and the sport’s ethos is a dynamic and ongoing interactive process learnt over time.

In Øydna and Bjørndal’s^39^ study of the culture of athlete development in Norwegian female handball, pervasive cultural beliefs regarding what constitutes appropriate training and development were associated with an overemphasis on the *amount* of training. Athletes exhibited individualised caution, denial or self-censorship to reduce the training load. Such self-expectations were found to affect the health and well-being of the athletes negatively:

I feel like I am failing myself, and that I am seen as ‘she who is always injured’. That is not how I want to be recognised. I want to be identified as ‘The handball player’, not as the one who always sits in the stands because she cannot train, and cannot play matches. For me, it is more about [what I think about] myself than what others think of me.^39^

In Wall *et al*’s^38^ investigation of athletes who reported sport-related lower back pain, cultural beliefs in sport, in turn, were found to influence the belief systems of the athletes:

I've been told by pretty much everyone that back pain is part of [sport], um, and that I’m just gonna have to live with it and that everybody gets a little bit of back pain. It’s just part of the sport.^38^

Collectively, these findings underscore how the ethos, rules, norms and expectations of sport shape athletes’ decision-making processes and lived experiences.

### Implications for developing complex interventions to reduce injuries and promote sustainable practice

This narrative review has identified and summarised how interpersonal and contextual dynamics influence the risk of youth sports injuries, place pressure on athletes and even lead them to compromise their health. This pressure often results in athletes, coaches and medical staff making inappropriate decisions and engaging in behaviours that may harm athletes. Many examples underscore the detrimental effects of sociocultural influences, particularly the normalisation of staying silent about injuries and the pressure to continue playing while injured. The findings from the included studies were observed across various youth age groups and performance levels. They highlighted the pervasive nature of these challenges and the complex interactions between coaches, athletes and medical staff. Moreover, insufficient coordination across different contexts, such as between school and club practice settings, exacerbates these challenges. It also significantly increases the risk of injuries and compromises athletes’ overall health and well-being. These findings have implications for developing complex interventions to foster healthier practices in youth sports.

Athletes who have coaches who underplay injuries are more likely to be willing to compete with injuries.^40^ This indicates that the health of youth athletes can be negatively affected by coach–athlete relationships and the pressures that coaches apply. Interventions that address the vulnerability of youth players are, therefore, essential, and positive coaching practices related to injury prevention and athlete well-being should be encouraged. Further, coach education programmes should include modules on the interpersonal and contextual dynamics that influence youth sports injuries and strategies for creating a supportive training environment.

It is worth noting that coach education content is often normative, decontextualised and fragmented. It is typically focused on individual coaching elements such as exercise physiology, sport psychology and training methodology rather than on the interpersonal relationships and contextual factors important for sustainable athlete development.^41^ Coaches seldom adopt a dynamic biopsychosocial perspective of athlete learning and development.^36 42^ A more comprehensive approach is essential, contextualising youth athletes’ experiences and behaviours, underscoring the important role of coaches and integrating diverse perspectives regarding injury prevention. The complexity of interpersonal relationships, including peer pressure and social support, makes it important that interventions focus on enhancing communication among athletes, coaches and medical staff. It is also important that interventions foster a culture of openness and support within sports teams and offer more suitable approaches to injury management.

The effects of mesosystem dynamics on athletes indicate that achieving a balance between academic and athletic commitments is important.^43^ The increasing pressure of sports commitments during adolescence may lead athletes to neglect other aspects of their lives, such as spending time with friends and family or doing other leisure activities that promote well-being.^30^ Hence, there is a need for closer collaboration between educational institutions and sports organisations to ensure well- and better-coordinated schedules that allow for sufficient athlete recovery time. Doing so is especially important given the increasing involvement of public actors (such as community, voluntary-based sports clubs and sports organisations) and private actors (such as sports schools and private football academies) in youth sports. This growing complexity underscores the importance of enhanced coordination across such settings, emphasising preventing athlete burnout, reducing injury risks and preserving athlete health and well-being. This coordination is essential for maintaining the quality of opportunities provided to youth athletes by sports organisations.

Contextual dynamics, such as cultural beliefs and norms within specific sports settings, significantly shape athletes’ behaviours and attitudes toward injuries.^21 44^ For example, youth athletes involved in elite sports systems, such as talent development pathways, are shown to be more willing to risk injuries and compromise their health.^45^ Reducing and preventing such risks necessitates context-specific interventions that emphasise a holistic approach to athlete development and a focus on reducing the pressures experienced by athletes within their wider social environment.

Our review identified instances where athletes lacked sufficient awareness or understanding of the consequences of pushing through injuries.^30^ We, therefore, recommend implementing educational programmes, both for athletes and parents, to promote a more proactive approach to injury management. These programmes should stress the importance of seeking professional advice when needed. Doing so can help athletes manage their injuries better before they escalate into more serious and career-threatening issues. It is also imperative that health management strategies not treat health problems as sudden events but recognise the important influence of psychological and sociological factors on athlete health.^36^

While constructive policies and guidelines are important, their effectiveness is limited without constructive, high-quality interpersonal relationships and positive coach-athlete care and support. Intense, sport-specific, year-round training and competition, coupled with the pressure of competing for selections, heighten the risk of injuries and should be avoided by sports organisations.^46^ Instead, appropriate demands and balanced competition schedules are needed. In addressing the quality of interpersonal relationships, coach education should prioritise the need for ethical and appropriate coaching practices.^47^ The examples in this review (and other relevant sources) of (in)appropriate interpersonal and contextual dynamics could be useful ways to educate coaches about best practices and help them to improve athlete health and well-being.

## Conclusion

This narrative review has identified a range of studies that explored how interpersonal and contextual dynamics shape athletes’ experiences of risk and injuries in youth sports. Notably, most of these studies were from European countries and, unusually in sports science research, included only female participants. Within the microsystems of interpersonal dynamics, coach–athlete relationships and the phenomenon of pushing through injury and pain emerged as significant factors that impact the health and well-being of athletes. Furthermore, dynamics within the mesosystem of adolescent athletes highlighted the delicate balance required between academic and athletic commitments to prevent burnout and reduce the risk of injury. Finally, the review highlighted that contextual dynamics shape athletes’ and coaches’ behaviours and practices and are deeply influenced by institutionalised competitive sport’s ethos, rules and norms. For example, the prevailing culture of masculinity (eg, competitiveness, hierarchy and dominance, suppression of emotions, and physical strength and toughness), coupled with the pressure to perform well and meet performance expectations (either self-imposed or from coaches and teammates), can lead athletes to take unnecessary risks or return to play before they have fully recovered from their injuries.

Developing appropriate complex interventions for injury prevention and sustainable sports practice in youth sports, as Skivington *et al*^10^ argue, requires the application of more qualitative methodologies that take a more comprehensive, contextual and inclusive approach. These methodologies can be integrated into developing interventions and enable the recognition of interpersonal and contextual dynamics, which, as the studies have shown, markedly influence athletes’ injury risk and injury experiences.^48^ Nurturing a culture that prioritises athlete well-being and values long-term health over short-term performance has the potential to contribute to safer and more sustainable sporting environments for young athletes.

## supplementary material

10.1136/bmjsem-2024-001964 online supplemental file 1

10.1136/bmjsem-2024-001964 online supplemental file 2

10.1136/bmjsem-2024-001964 Abstract translation 1 This web only file has been produced by the BMJ Publishing Group from an electronic file supplied by the author(s) and has not been edited for content.
