# Supplementary material for: Exploring the interplay of interpersonal and contextual dynamics in youth sports injuries: a comprehensive narrative review
Source: BMJ Open Sport Exerc Med. 2024 Jul 16;10(3):e001964. doi: 10.1136/bmjsem-2024-001964 (PMC11253767; doi:10.1136/bmjsem-2024-001964)
Supplement: online supplemental file 1 [file bmjsem-10-3-s001.pdf]

## Supplementary Material 1. Empirical findings organised by themes

| Study | Themes | Examples supporting themes |
| --- | --- | --- |
| Barker & Bailey [25] | Interpersonal dynamics | I told Dave about a knee injury. I had told him I was in pain, as he always has a go if you don't tell him before the session. But I always feel like I have to push through an injury for fear that he won't respect you as an athlete. I never have an issue with pushing, but then part of me feels he should be the one telling me to stop. At no point did he say stop, so I carried on … (p. 53) |
| Barker-Ruchti & Schubring [26] | Interpersonal dynamics | Upon returning from the second competition, Marie wanted to go home. Her coach, however, had other plans and ordered Marie to continue training. At that point, Marie could only see one way out: being injured. In her first training session, Marie hurt her ankle, which once a medical practitioner confirmed her inability to train, allowed her to leave Rauham. (p. 74)<br><br>Marie resorted to limiting her interaction with Teresa to technical training questions and tried to divert attention away from her weight. One way she did this was by concealing from her coach when she was hurting or injured, because Teresa notoriously related poor performance or pains to weight gain. (p. 74) |
| Bjørndal, Andersen & Ronglan [27] | Interpersonal dynamics | Honestly, if I had never been injured I would never have even thought of quitting. […] When I was injured for the first time and was through half a year of rehabilitation, I was very motivated. [...] But the next time, when I got the message I would be out for about a year... that is when those thoughts [about quitting] slowly arose. (p. 539“<br><br>"It came to the point," commented Vanessa, "where [the coaches/club] knew about [my chronic injury] and I just wished that they had told me to stop. It got to the point where I broke down in practice. I just fell to the ground and started screaming [because of the pain]." (p. 539)<br><br>I should not have played in three to four teams or skipped practice to play matches. But players do not say 'no' to playing a match instead of going to practice. The coaches need to be determined and pose a demand: that you actually need to practise if you are to play. I often went straight from being injured to playing matches. (p. 540)<br><br>Nobody took me seriously. I said that [the injury] hurt. … When I got back home, we were on a pre-season training camp and there [the coach] did not take me seriously. I told him that it hurt but he commanded me to run high-intensity intervals with the rest of the team. When I came back home, I was totally wrecked (p. 540) |
| Bjørndal & Ronglan [28] | Interpersonal dynamics | Now I've been injury free for a long time. When I played with three different age groups, I was injured a whole season because of my back. Then I became, at a very young age, conscious that I needed to become stronger. So now I'm really satisfied with my training schedule and that I get to train enough strength training and that it's not too much handball specific practice on court. I'm not saying that's why Cecilia and many others are injured … but I don't get the same training load from handball practice that many of the others have. (p. 12)<br><br>I didn't manage to hold back before, but now I've been injured so much that I've become much better at it. I now manage to control when I need to rest and when I can go all in. I kind of feel the difference between dangerous pain and pain that's just supposed to be there. (p. 12)<br><br>Last year, I thought it was difficult. I felt I was dodging practice because I was injured so much, and then I felt I had to participate in the school training sessions. Often it became two handball sessions a day. Today, I have a much better dialogue with the school coach. (p. 12) |

| | | |
|---|---|---|
| Kuhlin, Barker-Ruchti & Stewart [30] | Interpersonal dynamics | If I am sick or injured, will she [the coach] think that I am not dedicated enough? My coach is probably right about Michaela. If she wanted to become a figure skater, she should be here. (p. 13)<br><br>I have been in so much pain, but I have not missed a single training session. My parents have begged me to tone down the training, but I have refused to do this. Recently, my leg started to fold due to the pain. I cannot put weight on my right hip anymore. My coach put me in the corner to practice the one thing that worked – spins...While doing the spins repeatedly, I repressed the feeling of pain. It did not matter. (p. 16) |
| Schubring & Thiel [31] | Interpersonal dynamics | I hope that it will simply pass (laughs). Don't give up hope and it'll be ok. (p. 311)<br><br>Yes. Back. Well, I had a tear at lumbar vertebrae four five, and water built up. And you can't just draw it out. You have to just wait until it closes. Well, until the water comes out and it closes up again. And that's why it took so long. (p. 313)<br><br>I've told John [the coach] that I, for example during the mountain runs, that I don't want to run downhill. That I just want to go up, and then ride down on a bike. And that I just want to take it easy there. (p. 315)<br><br>Discipline too. So, now, when he says: 'okay go jogging at this time and this time', then I actually go jogging every time. (p. 316)<br><br>So there the doctor gave me a shot. And then I could do stuff for two days and then, after that, nothing again. So then, before quals, Jason [the supervising physician] injected me. Just at youth championships, because when we were abroad another doctor injected me. And he had arranged with Jason, somehow, what the shot was and when and so on. (p. 317) |
| Podlog et al. [35] | Interpersonal dynamics | It has been painful just because it's constant; I'd say more psychological. Because I knew what I'd done, I was really upset, but not because it was hurting. I knew I was going to miss out on Nationals and because I knew that I was missing that six month period of improvement. (p. 441)<br><br>My goal is to play, I suppose, for the rest of the year without re-injuring and at the grand final when we start to play again. (p. 442)<br><br>Heather reported "trying harder [in rehabilitation] because encouragement from her coach was motivating." (p. 442)<br><br>Being able to "play at my full potential, injury free, one hundred per cent" or simply "just being there" and being "able to play like everybody else does." (p. 443) |
| Cavallerio, Wadey & Wagstaff [29] | Contextual dynamics | I bend forward, placing my hands on my knees. I try to catch my breath, while my heart plays tug-and-war with my rib cage. "Oh no", I say under my breath. "It's back!" Soon I start to feel the pain crawling down my spine. Clearly, it didn't like all that heavy landing. "Leave me alone," I tell it. "I need to train!" My Mum wanted to keep me home today, but I refused. It's the Regional's next week. A 'good' gymnast doesn't miss a training session. (p. 103)<br><br>I pray to myself that Trudy will remember that my back has been hurting lately. Although I've rested my back this week for the Regionals, as Trudy told me to, the pain is still lurking around. I really hope it will go away soon. But I certainly don't feel like I can tell her now, after I've turned up late. It's too easy to imagine the conversation in my head. (p. 104)<br><br>The pain starts shooting up through the left side of my back, as if wanting to prove who is stronger, urging to stop me. I remind the pain that I am a gymnast, and I'll do what any 'good' gymnast would do: I grit my teeth and smile. (p. 104) |

| | | |
|---|---|---|
| | | "Ouch!" Again, the pain puts what feels like a knife in my back. I wish it would just go and leave me alone. With every movement it cuts deeper and deeper, radiating through my whole body. "Regionals, Sally, think of the Regionals", I tell myself. (p. 104)<br><br>I sigh, and tell the pain to please leave me alone for 5 minutes, just 5 minutes! Then, I'll rest. I promise I'll rest. Please, just 5 minutes. (p. 104)<br><br>The gymnasts closest to me see my tears and I sense they feel my pain. I can see in their eyes the desire to help, sharing a look that also tells me they can't, and they continue to practice their own routines. (p. 105) |
| Schubring & Thiel [32] | Contextual dynamics | Well, in the beginning, it didn't make a difference to me – it still hurt but I played because I absolutely wanted to play. That's it too. My ambition, that's really quite dumb, because I always want more than I should. (p. 86) |
| Fenton & Pitter [33] | Contextual dynamics | You know, there was a time I played through a little bit of a minor concussion, I´m, things like that. When you are in the heat of the wound you don't really [sic] because of the adrenaline you are running at. Sometimes you don't recognize some injuries even though they might be severe, because you're so worked up and you're basically running off adrenaline and surges... (p. 216)<br><br>Just like in games, if I jam my fingers or break my fingers, I've broken fingers and still had to play in the game; that's something if you still feel you can still play you have to keep it down so you don't get pulled off. (p. 216-217) |
| Malcom [34] | Contextual dynamics | "Fire it home! Come on, throw it in here!" Liz threw the ball hard, and it landed in Joe's hand with a smack. With a smile on his face, Joe said loudly, "That's it! THAT HURT. Good throw!" When Joe caught their hard throws in his bare hand without flinching, he demonstrated his "toughness" as a ballplayer and modeled the sport ethic to the girls on the softball team. (p. 509) |
| Thiel et al. [36] | Contextual dynamics | Well, withouth injuries...it doesn't work. It's IMPOSSIBLE that one...after so manye years has never had an injury or that everything goes fine. That's impossible...because the body is stranied too much...it CAN'T bear it. (...) Okay, it is simply part of it. (p. 244)<br><br>Okay, if one has broken his FINGER or has had a capsulation rupture, these are things, when you touch it, it is painful, but when it is taped or iced then you think that it is OKAT again. (p. 246)<br><br>At the end of the day it is like THAT: In competition, nobody is interested in whether you suffer pain or not. You don't get extra points for chenching your TEETH. Therefore you don't tell it to anybody or SHOW it but you try to play (laughs) the HARD guy. (p. 246) |
| Von Rosen et al. [37] | Contextual dynamics | I Had to do everything by myself. I had no help from my coach. It was just me and it felt like we never discussed injuries, even though it was common. Only my closest frields supported me. (p. 735)<br><br>Everybody gets injured and you have to accept the injury tp be able to continue with sports participation. (p. 735)<br><br>I started feeling pain when I started doing intensive training. But then I did not think that there was something wrong with experiencing pain. After a few years I realized that it was not normal to feel pain during sports. (p. 735) |
| Wall, McGowan, Meehan & Wilson [38] | Contextual dynamics | I've been told by pretty much everyone that back pain is part of [49], um, and that I'm just gonna have to live with it and that everybody gets a little bit of back pain. It's just part of the sport. (p. 74)<br><br>I didn't, I just didn't want to seem like I was … being lazy or I didn't want to just miss out over- miss out on the training or anything like that. So normally if I did feel a bit of back pain it would take me a while to say it or I wouldn't say it at all. (p. 74) |

| | | |
|---|---|---|
| | | I was really angry, upset, I was stressed I was just … it just didn't make any sense to me …. they [coaches] would act like they care but they just care about … winning and … their team, they don't really care about, …me as a person, or … anyone as a person I feel like. That's how it came off, at least. (p. 74) |
| Øydna & Bjørndal [39] | Contextual dynamics | Yeah, it can be very much like, 'Oh, you are injured all the time, that probably means that you don't do your strength training'. I feel like people are quick to trash talk [the injured players] and say things like 'Yeah, she is injured but can blame herself!'. And that creates an environment where it is not okay to be injured. (p. 10)<br><br>I feel like I am failing myself, and that I am seen as 'she who is always injured'. That is not how I want to be recognised. I want to be identified as 'The handball player', not as the one who always sits in the stands because she cannot train, and cannot play matches. For me, it is more about [what I think about] myself than what others think of me. (p. 11) |
