# Supplementary material for: Exploring the interplay of interpersonal and contextual dynamics in youth sports injuries: a comprehensive narrative review
Source: BMJ Open Sport Exerc Med. 2024 Jul 16;10(3):e001964. doi: 10.1136/bmjsem-2024-001964 (PMC11253767; doi:10.1136/bmjsem-2024-001964)
Supplement: online supplemental file 2 [file bmjsem-10-3-s002.pdf]

Supplementary Material 2. Overview of included studies (n=15)

Reference number	Authors, year and country	Methodology	Participants	Age	Sport	Study purpose	Unit of analysis	Themes and findings
25	Barker & Bailey 2016 United Kingdom	Interviews	N=1 Female	19	Distance running	To investigate the impact of key and cumulative events in an athlete's life on her engagement in distance running and with the coach with whom she worked	The unique life story of an individual athlete in relation to the coaching environment	Coaches wield considerable influence in shaping the identities and experiences of athletes. This underscores the importance of the coach-athlete relationship. In a poignant example, the athlete persevered through injury and discomfort, driven by the apprehension that her coach might not value her as an athlete if she didn't.
26	Barker-Ruchti & Schubring 2016 Sweden	Interviews	N=1 Female	N/A*	Artistic gymnastics	To explore an athlete´s experiences of moving into and out of this sport	Athletic identity	In sport, the learning process includes the development of athletes' self-perception through their interactions within the sporting context and with key figures, such as coaches. For example, compromising health may serve as a method of learning to set boundaries in the coach-athlete relationship. This embodiment of learning can persist over time and pose challenges when transitioning out of sports, as evidenced by difficulties in shedding a high-performance gymnastics identity in this particular instance.

27	Bjørndal, Andersen & Ronglan 2018 Norway	Interviews	N=9 Females	18-19	Handball	To explore successful and unsuccessful transitions in Norwegian handball, specifically the journey from the youth player level to the adult elite level.	Experiences of the transitions from a junior level to a senior elite level, and to the adult elite athlete level	Repeated injuries were caused by various factors, including the extensive number of activities in the talent development context, the high-risk nature of competitive handball, and because coaches were unable to adapt to each other’s constraints, mutually and sufficiently.
28	Bjørndal & Ronglan 2018 Norway	Interviews	N=12 Females	16-17	Handball	To investigate athletes’ experiences of moving toward playing at an adult elite level within a Scandinavian team sport setting.	Experiences of pursuing top-level play	Well-functioning coordination between different practice settings appears to be central to successful long-term talent development. In contrast, a breakdown in coordination may lead to insufficient recovery, and increase the risk of injury or burnout.
29	Cavallerio, Wadey & Wagstaff 2016 Italy	A 12-month ethnographic study, including participant observation, individual interviews, and focus group interviews	N=16 Females	10-15	Rhythmic gymnastics	To gain an in-depth understanding of overuse injuries in rhythmic gymnastics from a psychosocial perspective.	Experiences of overuse injuries	Cultural norms, values and behaviours play a pivotal role in shaping the incidence and encounters of overuse injuries. Sociocultural values such as the pursuit of sporting success, discipline, and the quest for perfection, coupled with social norms such as punctuality and respect for the coach, collectively contribute to a physical embodiment that can result in overuse injuries.
30	Kuhlin, Barker-Ruchti & Stewart 2020 Sweden	Auto-ethnographic	N=1 Female	N/A*	Figure skating	To explore the long-lasting impacts of the coach-athlete relationship on a former figure skater.	Experiences within the coach-athlete relationship	The athlete’s deep-seated identity in figure skating and her corresponding behaviours fostered a coach-athlete relationship that was characterised by trust, dependence and obedience. This dynamic hindered her awareness of the severity of her injury and the detrimental effects on her health, well-being, personal development and sporting career.

31	Schubring & Thiel 2014 Germany	Interviews, participant observation	N=8 Males (n=4), females (n=4)	14-18	Gymnastics, biathlon, handball, wrestling	To examine and identify: (a) how young athletes navigate challenging growth experiences in elite sport, and (b) the typical coping strategies employed by those affected.	Youth athletes' experiences in elite sport	This study reveals five typical coping strategies among youth athletes: distancing, rationalization, active agency, self-disciplining, and responsibility transfer. These coping strategies have different health-compromising side effects, such as ignoring pain, injury, and growth problems
32	Schubring & Thiel 2014 Germany	Interviews	N=8 Males (n=3), females (n=5)	15-17	Artistic gymnastics, biathlon, handball and wrestling	To explore challenging growth experiences.	Experiences of growth	Overuse and injury conditions often arise due to ongoing discrepancies between athletes' physical capabilities and the demands placed on them within their environment. A problematic interplay between biology and social factors is evident. The cultural, structural and biographical dynamics create challenging scenarios for adolescent athletes´ healthcare.
33	Fenton & Pitter 2010 Canada	Ethnography, including field notes, questionnaires, interviews	N/A Males	15-18	Rugby	To enhance an understanding of the social influences on pain and injury behaviour in sports. The objective of the study was to theorise and observe the dynamic interaction between social influences and the physical body.	Socialization processes	Social influences on pain and injury were primarily shaped by the rules of the rugby, by social learning (including influences from the coaches, trainers/medical staff, and teammates), and by athlete status within the sporting context.
34	Malcom 2006 USA	Three-year ethnographic study	N/A Females	11-16	Softball	To investigate the experiences of novice female soft ball players as they confront a specific sports culture regarding the management of minor injuries and pain.	Reactions to the cultural contradictions of female athleticism	The conflict between the traditional norms of femininity and the values of the sport ethos provided insights into the socialization process of young females. The study showed that the majority of girls conform to the expectations of the sport ethos by learning to cope with pain and injuries, resiliently disregarding them.
35	Podlog et al. 2013 Australia	Interviews	N=11 Males (n=3), females (n=8)	12-17	Basketball, netball, soccer, rowing, track and field	To examine the injury recovery and return-to-sport experiences of adolescent athletes.	Experiences during recovery from injuries and the process of returning to sport.	The study identified four key themes associated with athletes' injury recovery and return-to-sport experiences, namely: (a) injury (b) stress, (c) coping strategies,

								(d) experiences with social support, and (e) recovery outcomes. Additionally, the authors suggested that addressing competence and related issues, as emphasised in the basic psychological needs' theory (including proficiency, connectedness, and belonging) may be pertinent in the injury experience of adolescent athletes.
36	Thiel et al. 2015 Germany	Interviews	N=24 Males and females (the study provided no breakdown by gender)	14-18	Artistic gymnastics, biathlon, handball, wrestling	To examine the key moments in the ongoing development of adolescent elite athletes, and their experiences with health and ill-health.	Experiences of health and ill-health	Injuries and pain are common in elite sports, even at a young age. Athletes become accustomed to training and competing while enduring constant pain. To meet the specific demands of their sport, they develop behavioural patterns that, outside the context of elite sports, would be deemed harmful. Coaches play a crucial role in this process.
37	Von Rosen et al. 2018 Sweden	Focus group interviews	N=20 Males (n=8), females (n=12)	15-19	Athletics jumping, sprint, middle-distance running, golf, orienteering, bowling, cross-country skiing, downhill skiing, freestyle skiing	To examine the perception and experience of injury among adolescent elite athletes.	Perceptions and experiences of injury	The results highlighted the risk of young athletes experiencing a loss of self while injured.
38	Wall, McGowan, Meehan & Wilson 2023 Ireland and US	Interviews	N=9 Males (n=3), females (n=5), Undisclosed (n=1)	14-19	Rowing, cycling, hurling, Gaelic football, rugby, American football, basketball, lacrosse, cheerleading	To examine the experiences of adolescent athletes who have reported an episode of sport-related low back pain. This study: (a) examines the impact of this pain on daily life, (b) explores the effects it has on relationships with parent/guardians, teammates, and coaches, (c) examines athlete experiences of the management/treatment of low back pain, and (d) explores athletes understanding of low back pain	Experiences of sports-related low back pain	The results illustrate that the experience of low back pain is influenced by the prevalent culture of tolerating pain and injury in a sporting context.
39	Øydna & Bjørndal 2022 Norway	Focus group interviews, individual interviews	N=24 Females	17-19	Handball	To examine how the social interactions of youth handball players are intricately connected with the ideals, beliefs and norms	Experiences of social interactions, including ideals, beliefs and norms	A narrow emphasis on training quantity reflects a narrow understanding of the essence of youth athlete learning. The study

						associated with youth athlete learning in Norwegian handball. This examination involves an exploration of how these elements are communicated through coaching practices.		additionally proposes that problematic norms within sports are communicated through social pressures, experienced by athletes, and perpetuated through individualised caution, denial, or even self-censorship. These self-expectations are highly likely to have adverse effects on the health and well-being of athletes.

*Young adults reminisce about their time as adolescent athletes.
