# Supplementary material for: Exploring the interplay of interpersonal and contextual dynamics in youth sports injuries: a comprehensive narrative review
Source: BMJ Open Sport Exerc Med. 2024 Jul 16;10(3):e001964. doi: 10.1136/bmjsem-2024-001964 (PMC11253767; doi:10.1136/bmjsem-2024-001964)
Supplement: Abstract translation 1 [file bmjsem-10-3-s003.docx]

Skader i idrett og trening ses ikke lenger på som isolerte hendelser, men som resultat av komplekse faktorer. En idrettsutøvers helse påvirkes av dynamiske sammenhenger mellom prestasjon, biologi og de sosiale og kulturelle miljøene de er en del av. Til tross for denne forståelsen, har det vært lite fokus på hvordan relasjoner og miljøer kan påvirke skaderisikoen gjennom beslutninger som tas av trenere, foreldre og utøvere.

Denne gjennomgangen av forskningslitteraturen kombinerer innsikter fra sosiokulturelle studier i idrett med funn fra idrettsskadeforskning. Målet er å identifisere og oppsummere hvordan mellommenneskelige og kontekstuelle faktorer påvirker risikoen for skader blant unge idrettsutøvere.

Resultatene viser at utøvere ofte opplever press som kan gå utover helsen deres. Gjennomgangen fremhever viktigheten av å utvikle komplekse tiltak og strategier for å fremme sunnere praksis i ungdomsidrett. Spesielt bør tiltak fokusere på å øke bevisstheten om skaderisiko, utvikle gode kommunikasjonsferdigheter og skape støttende treningsmiljøer.
